# Combination Analysis of a Radiomics-Based Predictive Model With Clinical Indicators for the Preoperative Assessment of Histological Grade in Endometrial Carcinoma

**DOI:** 10.3389/fonc.2021.582495

**Published:** 2021-06-21

**Authors:** Tao Zheng, Linsha Yang, Juan Du, Yanchao Dong, Shuo Wu, Qinglei Shi, Xiaohan Wang, Lanxiang Liu

**Affiliations:** ^1^ Department of Magnetic Resonance Imaging, Qinhuangdao Municipal No. 1 Hospital, Qinhuangdao, China; ^2^ Department of Intervention, Qinhuangdao Municipal No. 1 Hospital, Qinhuangdao, China; ^3^ Scientific Clinical Specialist, Siemens Ltd., Beijing, China

**Keywords:** endometrial carcinoma, histological grade, radiomics, apparent diffusion coefficient, nomogram

## Abstract

**Background:**

Histological grade is one of the most important prognostic factors of endometrial carcinoma (EC) and when selecting preoperative treatment methods, conducting accurate preoperative grading is of great significance.

**Purpose:**

To develop a magnetic resonance imaging (MRI) radiomics-based nomogram for discriminating histological grades 1 and 2 (G1 and G2) from grade 3 (G3) EC.

**Methods:**

This was a retrospective study included 358 patients with histologically graded EC, stratified as 250 patients in a training cohort and 108 patients in a test cohort. T2-weighted imaging (T2WI), diffusion-weighted imaging (DWI) and a dynamic contrast-enhanced three-dimensional volumetric interpolated breath-hold examination (3D-VIBE) were performed *via* 1.5-Tesla MRI. To establish Model^ADC^, the region of interest was manually outlined on the EC in an apparent diffusion coefficient (ADC) map. To establish the radiomic model (Model^R^), EC was manually segmented by two independent radiologists and radiomic features were extracted. The Radscore was calculated based on the least absolute shrinkage and selection operator regression. We combined the Radscore with carbohydrate antigen 125 (CA125) and body mass index (BMI) to construct a mixed model (Model^M^) and develop the predictive nomogram. Receiver operator characteristic (ROC) and calibration curves were assessed to verify the prediction ability and the degree of consistency, respectively.

**Results:**

All three models showed some amount of predictive ability. Using ADC alone to predict the histological risk of EC was limited in both the cohort [area under the curve (AUC), 0.715; 95% confidence interval (CI), 0.6509–0.7792] and test cohorts (AUC, 0.621; 95% CI, 0.515–0.726). In comparison with Model^ADC^, the discrimination ability of Model^R^ showed improvement (Delong test, P < 0.0001 for both the training and test cohorts). Model^M^, established based on the combination of radiomic and clinical indicators, showed the best level of predictive ability in both the training (AUC, 0.925; 95% CI, 0.898–0.951) and test cohorts (AUC, 0.915; 95% CI, 0.863–0.968). Calibration curves suggested a good fit for probability (Hosmer–Lemeshow test, P = 0.673 and P = 0.804 for the training and test cohorts, respectively).

**Conclusion:**

The described radiomics-based nomogram can be used to predict EC histological classification preoperatively.

## Introduction

Endometrial carcinoma (EC) ranks sixth in terms of both morbidity and mortality amongst malignancies that affect women worldwide, with 320,000 new cases and 90,000 deaths occurring per year (1). Traditionally, the incidence of the disease has been higher amongst postmenopausal women, although, more recently, a trend of increasing disease rates amongst younger women has been observed (2). Studies to date have shown that, in addition to the tumor stage, the pathological grade of the tumor is also one important factor influencing its treatment. Conservative therapy with progestins can be adopted only when the disease is confined to the endometrium and the cancer is a well-differentiated [histological grade 1 (G1)] endometrioid adenocarcinoma (3). Less than 1.4% of low-risk EC cases [G1 and histological grade 2 (G2)] exhibited lymph node metastasis, while the rate of lymph node metastasis increases to nearly 6.4% amongst high-risk EC cases [histological grade 3 (G3)] (4). The 2009 International Federation of Obstetrics and Gynecology (FIGO) staging system did not consider the role of histopathological types in patient surgical plan and prognosis. Therefore, the system is limited in risk assessment for some non-endometrioid EC (5). To overcome this deficiency, the 2014 FIGO guidelines were revised to suggest that non-endometrioid EC should be treated as G3 tumors with para-aortic lymph node dissection (6). In 2015, the European Society of Medical Oncology also recommended that lymph node dissection should not be performed in low-risk patients (G1 or G2, with muscular invasion ≤50%), while systematic pelvic and para-aortic lymph node dissection should be recommended in high-risk patients (G3, with muscular invasion >50%) (7). While dilatation and curettage (D&C) or hysteroscopy can suggest the histological grade before surgery, such invasive examinations are painful, carry risks of bleeding and infection and still exhibit a certain probability of missed diagnosis or misdiagnosis; thus, the final accurate degree of tumor pathological differentiation is determined surgically (8–10). In addition, more importantly, the result of D&C is greatly affected by the operator’s experience which may result in an inadequate surgical resection. Developing non-invasive methods to accurately determine tumor grade before surgery would be of great significance, helping to alleviate patients’ pain, facilitate surgical planning in advance and reduce rates of under- and overtreatment.

While conventional magnetic resonance imaging (MRI) can assist with determining the presence and depth of the muscular infiltration of EC, its capacity to predict the preoperative histological grade of a tumor is limited (11). In the past 10 years, advances in radiomic technology have made it possible to deeply explore the biological nature of images and make up for the deficiency of subjective observation. Through a large amount of data extracted from medical images and high-throughput quantitative analysis, high-fidelity target information can be compiled to comprehensively evaluate tumor heterogeneity in space and time (12). An increasing number of scholars are paying attention to imaging radiomics, which has been widely used in the preoperative diagnosis, grading, treatment sensitivity assessment and postoperative survival prediction in patients with brain stromal tumors, lung cancer, colorectal cancer and nasopharyngeal cancer, thus accelerating clinical and translational research in oncology (13–16). Previous researchers extracted and analysed the radiomic features of MRI unenhanced as well as enhanced MRI and postulated that the omics parameters could be used to accurately assess both lymph node metastasis and lymph vascular space invasion (LVSI) in EC, supporting radiologists’ efforts in making a correct diagnosis (17, 18). Diffusion weighted imaging (DWI) is a functional imaging technique that has been studied for histological assessment of tumors and there have been studies combining the role of DWI and diffusion tensor imaging (DTI) for preoperative prediction of the tumor pathology (19). In the present study, we extracted and selected radiomic features from T2-weighted imaging (T2WI); ADC mapping and arterial, venous and delayed dynamic contrast-enhanced (DCE)-T1WI images; established a nomogram by combining carbohydrate antigen 125 (CA125) and body mass index (BMI) and compared such with DWI-based ADC model. With this, we ultimately aimed to establish a more accurate prediction model for EC histological grading that can provide support for treatment method selection.

## Materials and Methods

### Subjects

**Figure 1:**
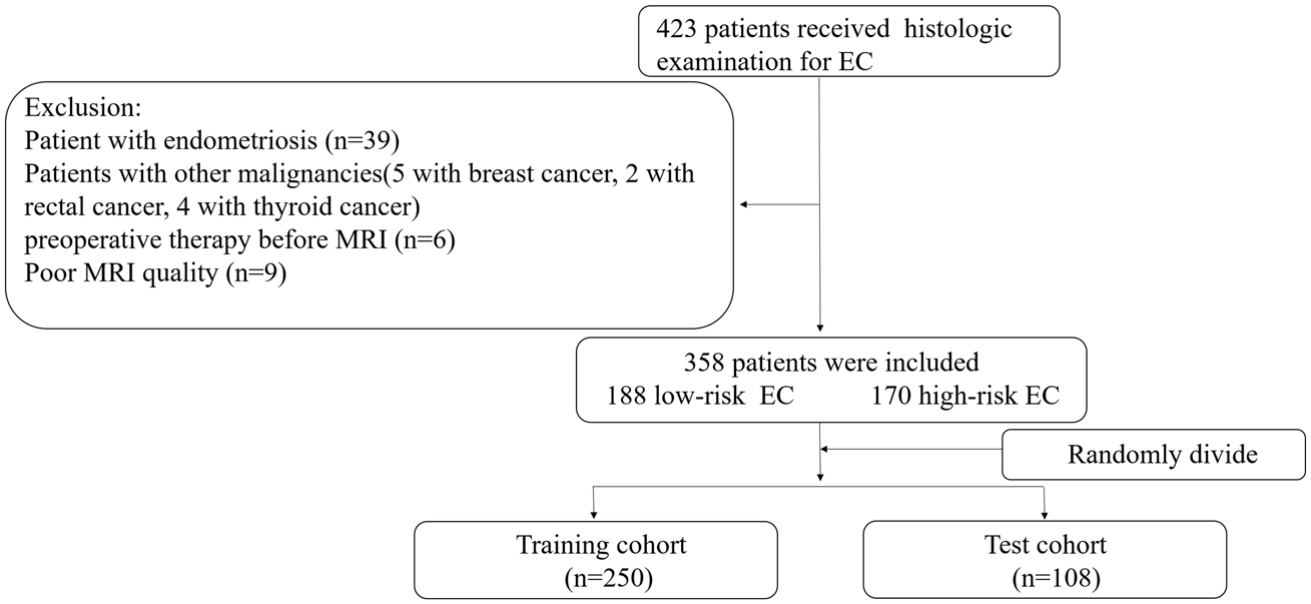
Recruitment pathway for patients in this study. EC, endometrial cancer; Low-risk= G1+G2, high-risk= G3 and non-endometrial carcinoma.

This retrospective study that incorporates anonymous data was approved by the Ethics Committee of our hospital and the need for informed consent was waived. A total of 421 cases of EC diagnosed by pathology assessments after surgical resection seen at our hospital from January 2010 to January 2020 were identified as initially eligible for inclusion. All patients underwent MRI plain and DCE assessments before surgery. All patients with EC underwent postoperative tissue differentiation classification (i.e., stratification as G1, G2 or G3, as detailed below). The inclusion criteria for this study were as follows: (1) having undergone MRI within two weeks before tumor resection and (2) did not receive chemoradiotherapy or targeted therapy before MRI. The exclusion criteria were: (1) presence of endometriosis or submucosal myoma regardless of whether the parameter measurement was affected, (2) presence of other malignant tumors and (3) presence of serious MRI image artefacts affecting the ability to perform parameter measurement. Finally, 358 patients were included in this study and were divided into the training (n = 250) and test (n = 108) cohorts according to a randomisation method at a ratio of 0.7 to 0.3 (**Figure 1**).

### MRI Examination

In this study, pelvic MRI scans were acquired on a 1.5-Tesla Siemens Avanto MRI system (Siemens, Munich, Germany) equipped with an eight-channel body coil. Patients were asked to fast 4 to 6 hours prior to imaging and void before the scan to reduce motion artefacts. The scanning area ranged from the antero-superior iliac spine to the symphysis pubis. The MRI protocol included the pelvic sagittal, coronal, and axial oblique views (perpendicular to the long axis of the uterus). The scanning sequence included the sagittal, coronal, and axial fat-saturation T2WI; axial DWI and axial three-dimensional (3D) volumetric interpolated breath-hold examination (3D-VIBE); sagittal 3D-VIBE. DWI was acquired by echo-planar imaging (b-value = 0, 800 s/mm^2^). During the axial and sagittal 3D-VIBE scan, the patient was asked to hold their breath at the end of the exhaled condition to reduce the collection of breathing movement artefacts. Before the contrast agent gadolinium diethylenetriamine penta-acetic acid (Gd-DTPA; Bayer Healthcare Pharmaceuticals, Berlin, Germany) was injected, the axial mask image was scanned; thereafter, Gd-DTPA was injected into the cubital vein using a high-pressure syringe (Spectris MR injection system, Medrad Inc., Warrendale, PA, USA) at a dosage of 0.2 mmol/L/kg. Axial images in the arterial, venous and delayed phases were collected at 25, 60 and 180 seconds after the injection of Gd-DTPA. The sagittal 3D-VIBE images were collected after the collection of images in the delayed phases. The specific MRI parameters are shown in **Table 1**.

**Table 1:** MRI protocol for GIST.

Sequences Parameters	MRI Sequences					
	Sagittal-T2WI	Coronal-T2WI	Axial-T2WI	Axial-DWI	Axial-3D-VIBE	Sagittal-3D-VIBE
Fat saturation	Yes	Yes	Yes	Yes	Yes	Yes
TR/TE (msec)	4340/92	4340/92	4340/92	75/2.38,4.79	4.44/2.16	4.44/2.16
Angle (°)	150	150	150	70	10	10
Slice thickness (mm)	4	4	4	4	3	3
FOV (mm^2^)	280	280	280	280	280	280
Voxel Size (mm^3^)	0.6×0.6×4.0	0.6×0.6×4.0	0.6×0.6×4.0	1.6×1.6×4.0	0.6×0.6×3.0	0.6×0.6×3.0
Interslice gap	10%	10%	10%	10%	0	0
Delay (s)					0, 25, 60, 180	
Scan time (s)	145	145	145	130	17	17
b-Value (s/mm^2^)			0, 800			

FOV, field of view.

### Histological Diagnosis

EC is primarily graded by the tumor architecture, with those having 5% or less of solid growth considered to be G1 tumors, those with between 6% and 50% of solid growth considered to be G2 tumor and those with more than 50% of solid growth considered to be G3 tumors (20, 21). G1 and G2 endometrioid adenocarcinomas were classified as low-risk EC, while G3 or non-endometrioid carcinomas (e.g., clear cell adenocarcinoma, serous adenocarcinoma) were classified as high-risk EC, suggesting a poorer prognosis (22).

### Clinical Data

The clinical indicators in this study for analysis were age, BMI, and serum CA125 levels. In clinical practice, BMI is quickly calculated to make an initial assessment of whether a patient is overweight or obese and people focus more on the presence or absence of an abnormal BMI, rather than its specific value. Therefore, BMI was converted into ranked data in this study. Normal weight was suggested by a BMI of 18.5–24 kg/m2, overweightness by a BMI of 24–28 kg/m^2^ and obesity was by a BMI of greater than 28 kg/m^2^. Low-weight patients with BMIs of less than 18.5 kg/m^2^ were not included in this study as this may have been due to cachexia caused by malignant tumors. The CA125 level was detected by chemiluminescence microparticle immunoassay (Cobas 8000 E602; Roche Holding AG, Basel, Switzerland). Univariate analysis, using a chi-squared test or student’s t test respectively, was conducted to assess differences in BMI and CA125 between the two groups. The analysis was performed in the X&Y software (X&Y Solutions, Inc., Boston, MA, USA).

### 3D Segmentation and Radiomic Feature Extraction

**Figure 2:**
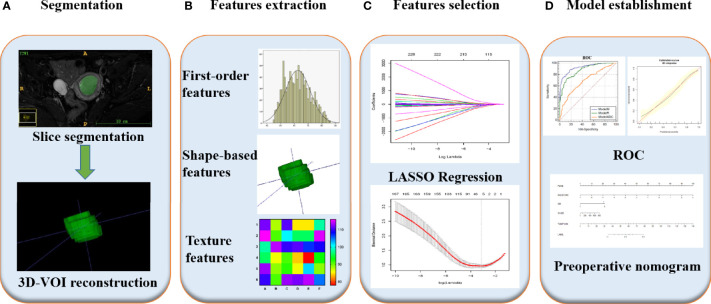
Radiomics workflow of model construction. **(A)** MR images segmentation. First, the boundary of tumor was delineated layer by layer on each image, considering necrosis, cystic lesions and bleeding areas inside the tumor during the delineation and 3D segmented tumor images were finally obtained using ITK-SNAP. **(B)** Radiomics features extraction. According to the segmentation image, a total of 100 parameters of 6 types were extracted from each set of images. **(C)** Radiomics features selection. After the parameters were normalized and dimensionality reduced, the characteristic parameters were selected and classified by LASSO regression. **(D)** Model establishment. Combined with two clinical indicators of location and size, nomogram was developed to establish a preoperative evaluation model, and its diagnostic efficacy was evaluated by ROC analysis.

First, images in Digital Imaging and Communications in Medicine format were downloaded from the PACS system of our hospital for analysis. Subsequently, the ITK-SNAP software (https://www.itksnap.org, version 3.6.0) was applied by two radiologists with five years of pelvic MRI diagnosis experience each who were not aware of the histological grading and clinical data of the tumor under review. The boundary of each tumor was delineated layer by layer on each image, considering necrosis, cystic lesions and bleeding areas inside the tumor during the delineation and 3D segmented tumor images were finally obtained. We then saved the segmented images and import them into the Pyradiomics toolkit (https://www.pypi.org/project/pyradiomics/, version 3.0), which was designed to facilitate the extraction of radiomic features running in the Python environment (https://www.python.org, version 2.7.0). The extracted radiomic features included first-order features (n = 18), grey-level co-occurrence matrix features (n = 22), grey-level dependence matrix features (n = 14), grey-level run-length matrix features (n = 16), grey-level size-zone matrix features (n = 16) and shape-based features (n = 14), for a total of 100 radiomic parameters. **Figure 2** presents a T2WI image of a patient with a low-risk EC profile as an example and describes the extraction process of the relevant radiomic features. More information about the texture feature-extraction methodology can be found in **Supplementary Method S1**. Next, the intra-class correlation coefficient (ICC) of the extracted radiomic parameters assigned by the two radiologists were calculated to evaluate the agreement of the data. An ICC of greater than 0.75 was considered to suggest good agreement.

### Statistical Analyses

#### Establishment of the ADC Model

On the postprocessing workstation of the Siemens MRI system (Leonardo 3682), ADC values of tumor parenchyma were manually measured by outlining circular regions of interest (ROI). Based on the actual size of tumors, the size range of ROI was 2.3cm^2^ to 14.1cm^2^ and the average was 6.1cm^2^ (**Supplementary Figure 1**). Special attention was given to avoid areas of cystic degeneration, necrosis and bleeding while including as much tumor parenchyma as possible. The above ADC value measurements were performed by the two radiologists mentioned above in section 2.5 of Materials and methods. Intra-group correlation coefficients (ICC) of the measurements were calculated after the procedure. An ICC of greater than 0.75 was considered to suggest good agreement. Univariate analysis, using a student’s t test, where appropriate, was conducted to assess differences in ADC values between the two groups. A binary logistic regression analysis was subsequently applied to build the ADC value model. The analysis was performed in the X&Y software.

#### Selection of Radiomic Features and Establishment of the Radiomic Model

The ‘normalise’ module in the FeAture Explore program (FAE, https://github.com/salan668/FAE, version 0.2.2) on Python (https://www.python.org; version 3.5.4) was used to normalise all radiomic parameters and thus eliminate the impact of the magnitude difference between different parameters to make the subsequent analysis results more reliable. Specifically, ‘normalise to unit with 0-centre’ was used to normalize the data in order to reduce large differences in the values of the different radiomics characteristics. Then, Pearson’s correlation coefficient was calculated. When the coefficient is larger than the threshold value (currently the default is 0.86), one of them is removed randomly. Through this method, dimensionality was reduced and similar characteristic parameters were removed. The methods of normalisation and dimensionality reduction adopted in this study are reported in the **Supplementary materials** (**Supplementary Method S2**). Subsequently, the least absolute shrinkage and selection operator (LASSO) regression module provided by the X&Y software based on the R software (https://www.r-project.org, version 3.4.3; The R Foundation for Statistical Computing, Vienna, Austria) was used for selecting the radiomic features most closely related to tumor histological grading. The complexity of LASSO regression model is controlled by the parameter λ. The larger the λ, the more refined the model is. λ is screened by 10 folds cross-validation. In the cross-validation method, the data will be divided into 10 equal fractions. First, the whole data will be fit and lambda sequence will be generated. Then, one fraction will be excluded each time, and the remaining nine fractions will be used for validation, and the average and standard deviation of any error acquired in 10 validations will be calculated. Finally, two models are produced. One is based on λ_min_, that is, λ when the mean value of the error is the minimum; the other is based on λ_1-SE_, that is, the maximum lambda of the error mean within 1 standard error of the minimum value. In this study, we chose the latter as the final model, because the latter included fewer radiomic parameters and the model was more refined. Based on the regression coefficient of the LASSO model and selected parameters, the Radscore of the training and test cohorts was calculated and the radiomic model (Model^R^) was established based on the Radscore of the training cohort.

#### Development of a Radiomic Nomogram and Comparison of Different Models

A receiver operator characteristic (ROC) curve was drawn by MedCalc (https://www.medcalc.org/, version 18.9; MedCalc Software BVBA, Ostend, Belgium). The area under the curve (AUC) was then adopted to compare the diagnostic efficacy between the ADC, radiomic and hybrid models, respectively. Further, differences in the AUC values between the three models were assessed using the Delong test (23). After combining the Radscore with BMI and CA125, logistic regression analysis could be carried out to establish a mixed model (Model^M^). Thereafter, we developed the nomogram of the mixed model to provide a quantitative guidance tool for the clinical diagnosis of tumor classification. The calibration of the radiomics-based nomogram was assessed using calibration curves. The calibration effect was evaluated with the Hosmer–Lemeshow test. Also, internal validation was performed with bootstrapping to correct for optimism of the model (24).

## Results

### Clinical Characteristics

Amongst the study findings, there were no significant differences in the distribution for age, BMI or CA125 amongst low- and high-risk cases in the two test cohorts (i.e., all P_a_ and P_b_ values were greater than 0.05), supporting the randomness and equilibrium of data allocation between the two cohorts. Patient characteristics in the training and test cohorts, respectively, are provided in **Table 2**. There was no significant difference in age between low- and high-risk patients (P = 0.517 and P = 0.729 for the training and test cohorts, respectively). However, the BMI distribution was different, with the proportions of overweight and obese individuals in the high-risk group being significantly higher than those in the low-risk group (P < 0.0001 for the training and test cohorts) (**Table 2**). Further, the serum CA125 level of high-risk patients was higher than that of the low-risk patients (P < 0.0001 and P = 0.035 for the training and test cohorts, respectively).

**Table 2:** Patient characteristics in the training and test cohorts.

Characteristics	Training cohort (*n* = 250)			Test cohort (*n* = 108)				
	G1&G2 (*n* = 132)	G3 (*n* = 118)	*P*	G1&G2 (*n* = 57)	G3 (*n* = 51)	*P*	*P_a_*	*P_b_*
Age (years)			0.517			0.729	0.481	0.450
Mean± SD	58.8 ± 13.2	57.7 ± 13.6		60.3 ± 13.9	59.4 ± 12.9			
Range	37-82	35-80		37-80	36-79			
BMI			**<0.0001**			**<0.0001**	0.346	0.117
Normal	92	36		43	16			
Overweight	31	94		13	29			
Obesity	9	6		1	6			
CA125			**<0.0001**			**0.035**	0.472	0.268
Mean ± SD	162.6 ± 184.6	327.4 ± 272.9		184.4 ± 203.4	277.8 ± 249.7			
Range(median)	0-570 (75.5)	0-779 (358.5)		0-590 (119)	0-779 (291)			

The P_a_ was derived from the student t or chi-square test of G1&G2 groups between training and test cohort and the P_b_ was derived from that of G3 groups between training and test cohort. Bold type indicates statistically significant difference.

### Diagnostic Performance of the ADC Value

**Figure 3:**
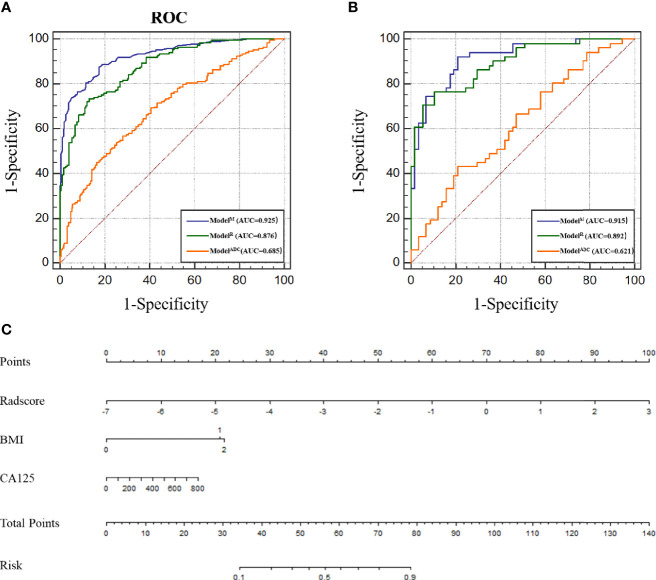
**(A)** Receiver operating characteristic (ROC) of different models in the training cohort. **(B)** ROC of different models in the test cohort. **(C)** Nomogram for predicting risk classification of EC. The nomogram was built in the training cohort with the Radscore, BMI and CA125. The probability of each predictor can be converted into scores according to the first scale points at the top of the nomogram. After adding up the scores of these predictors in total points, the corresponding prediction probability at the bottom of the nomogram is the malignancy of the tumor.

The ICC of the ADC value as measured by the two radiologists was approximately 0.963–of note, this value is greater than 0.75, suggesting good consistency between the two testers. Meanwhile, the ADC values of the high-risk populations in the training and test cohorts were 0.85 ± 0.38 and 0.90 ± 0.32, respectively, which were significantly lower than those of the low-risk populations in the two cohorts (1.14 ± 0.38 and 1.05 ± 0.32; P < 0.0001 and P = 0.015). The identification ability of the ADC value to discern EC cases of different pathological risk levels was evaluated by ROC curve drawing. The AUCs of the ADC value were 0.715 [95% confidence interval (CI), 0.6509–0.7792; sensitivity, 61.0%; specificity, 74.2%; **Supplementary Table 1**] and 0.621 (95% CI, 0.515–0.726; sensitivity, 43.1%; specificity, 79.0%; **Supplementary Table 2**) for the training and test cohorts, respectively (**Figure 3**).

### Diagnostic Performance of the Radiomic Features

**Figure 4:**
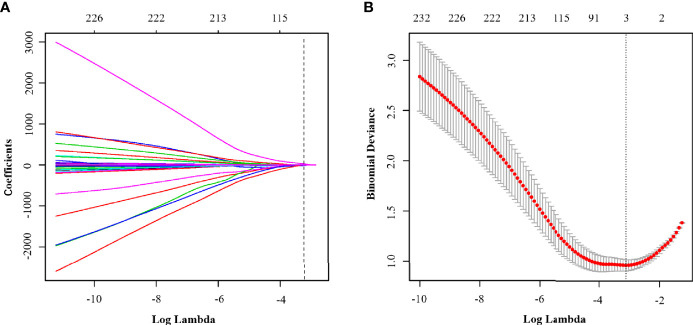
**(A)** LASSO coefficient profiles of the clinical parameters in Model^R^. **(B)** Binomial deviance profiles of the texture features in Model^R^. According to the 1 standard error of the minimum criteria (the 1-SE criteria), a log(λ) value of −3.107 was chosen (10-fold cross-validation, 1-SE criteria).

We extracted radiomic features from the final study group of 358 patients, with each having 5 sets of different MRI sequence images and with 500 parameters extracted from each patient. An ICC value of less than or equal to 0.75, which is considered to suggest poor parameter stability, should generally not be included in the regression equation analysis. According to this standard, a total of 190 parameters were excluded. Amongst the remaining 310 parameters, according to the 1 standard error of the minimum criteria (the 1-SE criteria), a log(λ) value of −3.107 was chosen (10-fold cross-validation, 1-SE criteria). Then the following three radiomic features with nonzero coefficients were deemed by LASSO regression to be of value in the tumor classification (**Figure 4**): LargeDependenceLowGreyLevelEmphasis@Venous, Maximum2DDiameterColumn@ADC and LowGreyLevelZoneEmphasis@ADC. According to the coefficients of the LASSO regression, the equation can be realised as follows:

Radscore = −1.3103 * LargeDependenceLowGreyLevelEmphasis@Venous + 0.03765 * Maximum2DDiameterColumn@ADC + 866.53184 * LowGreyLevelZoneEmphasis@ADC. The radiomic model was established according to the Radscore and the ROC curve was drawn. Here, the AUC of the model was 0.870 (95% CI, 0.828–0.913; sensitivity, 72.0%; specificity, 85.6%; **Figure 3** and **Supplementary Table 1**). In comparison with Model^ADC^, per the Delong test, it was found that the AUC of Model^R^ was greater than that of Model^ADC^ (P < 0.0001 for the training and test cohorts).

### Radiomic Nomogram Construction and Comparing the Performance of the Different Models

**Figure 5:**
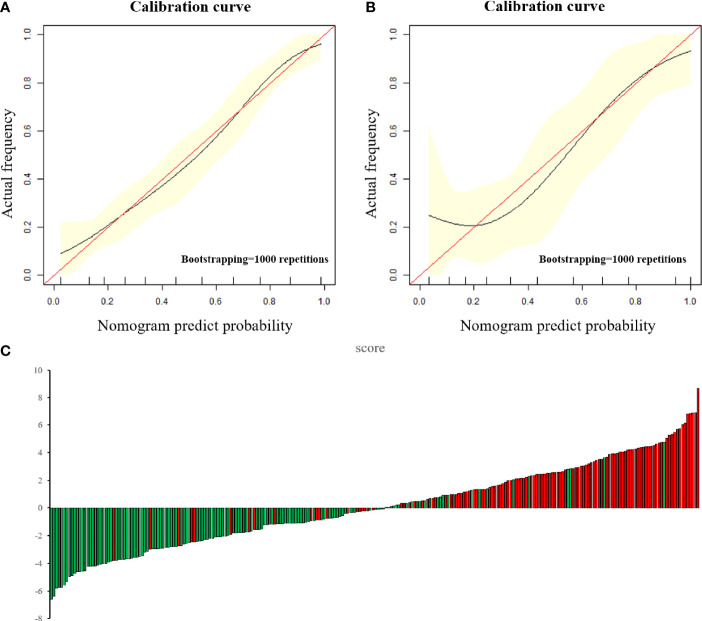
**(A**, **B)** The calibration curve in the training cohort **(A)** and test cohort **(B)**. The calibration curve depicted the agreement between the predicted risk classification score and the actual results confirmed by confirmed by examination. The red line represents an ideal prediction, and the black line represents the predictive performance. The closer the fit of the black line to the ideal line, the better the prediction. **(C)** Patient risk classification score output, while green bars show scores for those who were in low-risk group and red bars show that in high-risk group.

According to the above results, BMI and CA125 may constitute independent risk factors for predicting the histological risk. These two clinical indicators were combined with the Radscore to establish Model^M^ and the nomogram was drawn. The AUC for the training cohort was 0.925 (95% CI, 0.898–0.951; sensitivity, 88.8%; specificity, 81.5%; **Supplementary Table 1**), which was greater than that of the radiomic model. However, in the test cohort, there was no statistically significant difference between the AUC of Model^M^ and Model^R^ (P = 0.317), though this outcome may be related to the small sample size of the test cohort. The difference between the predicted results of Model^M^ and the gold standard was evaluated by plotting the calibration curves of the training and test sets (**Figure 3**). The calibration curves suggested good fitness for probability (Hosmer–Lemeshow test, P = 0.673 and P = 0.804 for the training and test cohorts, respectively). **Figure 5** presents the tumor risk classification score calculated by the model and suggests that it has a good level of ability to classify the low-risk and high-risk EC.

## Discussion

### The Ability of ADC Alone to Predict the Histological Risk of EC Is Limited

Although the ADC value of the high-risk group was lower than that of the low-risk group in this study, it was found by ROC analysis that Model^ADC^, which was constructed by applying the ADC value alone, exhibited only limited efficiency in predicting the EC histological grade. The ADC value is a functional imaging indicator commonly employed clinically to reflect the diffusion of water molecules in tissues and it has been widely adopted for the assessment of pathological grades of breast cancer, rectal cancer and other tumors (25, 26). Typically, the lower the degree of differentiation of tumor tissues, the larger the nucleus, the greater the number of organelles, the more obvious the nuclear atypia and the larger the nucleo-plasmic ratio. In addition, the cells of high-risk tumors are larger, more numerous and more densely packed than those of low-risk tumors. The above factors are believed to culminate in the restriction of the diffusion movement of water molecules inside and outside poorly differentiated tumor cells.

One previous study suggested that the combination of whole-tumor volume and ADC can be used for predicting tumor grade (27). However, the diagnostic value of DWI with quantitative analysis of ADC remains controversial. Rechichi et al. observed that the ADC value in EC did not display a significant relationship with tumor grade, depth of myometrial invasion or presence of lymph node metastasis (28). The ADC value can only reflect the average water diffusion in a tumor and the underuse of complex signal information from within the tumor tissue leads to an insufficient understanding of the heterogeneity in the tumor. In this study, we found that adopting ADC alone is less effective in predicting its classification. Therefore, to successfully dig deep into a large amount of imaging data gleaned from inside the tumor *via* different angles and to improve the ability to identify EC of different risk levels, radiomics was adopted in this study to analyse ADC, T2WI and DCE-T1WI MRI scans.

### Radiomics Can Better Reveal the Histological Difference of EC When Compared With ADC

Current imaging radiomic technology can automatically identify and extract medical image features and transform them into image feature data that can be mined through automated high-throughput algorithms. Although radiomics operates from a more macroscopic perspective than genomics or histological markers, the indicators in this field remain good indicators of intra-tumor heterogeneity (29). Heterogeneity is an important biological characteristic of malignant tumors and manifests as inconsistencies in tumor cell density, microvascular density, cell proliferation and apoptosis. Tumor heterogeneity occurs due to changes in the tumor microenvironment caused by mutations in malignant tumor genes, which not only lead to abnormal cell proliferation and apoptosis but also to the appearance of abnormal vascular structures (30). Abnormal tumor angiogenesis may result in hypoxia in tumor areas, increase the local stromal hydrostatic pressure and raise the risks of tumor invasion and metastasis (31). Therefore, radiomics is a potential method to predict the histological grade and prognosis of tumors.

In this study, 3 features were finally determined from amongst 310 features by LASSO regression to have the closest relationship with tumor risk: LargeDependenceLowGreyLevelEmphasis@Venous, Maximum2DDiameterColumn@ADC and LowGreyLevelZoneEmphasis@ADC. One of the above three indicators is related to venous-phase enhancement of the tumor, while the other two are related to ADC images. LargeDependenceLowGreyLevelEmphasis@Venous measures the joint distribution of large dependence with lower grey-level values. The larger the parameter, the more heterogeneous the signal may be in the venous enhancement image. In addition to being able to characterise the relationship between the tumor and its surrounding structure, previous studies have shown that dynamic enhanced scanning for EC can help identify its pathological risk. The maximum enhancement degree of G1 differentiation EC was significantly higher than that of G3, indicating that the nature of low-risk tumor was similar to normal endometrium, with low cell density and abundant glands and blood vessels. Maximum2DDiameterColumn@ADC is a shape-based feature related to tumor diameter measured on the ADC map. The larger the parameter is, the larger the tumor diameter is. Tumor size is known to be related to tumor proliferation rate and higher histological grading may result in faster tumor proliferation rate and therefore larger tumor size (32). Previous studies have shown that, compared with T2WI and T1WI, the EC measured on the ADC map is the closest to the true size of the tumor, which may be due to the fact that the signals of dilating blood vessels and myometrium around the tumor are well inhibited in the DWI and ADC map, therefore showing the tumor boundary most clearly (33). Due to this advantage, in addition to distinguishing the risk of the tumor, ADC value is also one of the important indicators to determine whether EC has myometrial invasion or not (34). The LowGreyLevelZoneEmphasis@ADC relates to the lower grey-level size zones inside the tumor: the greater the value, the larger the lower grey-level zones inside the tumor may be. As compared with the ADC value that reflects the average diffusion of water within a tumor, this value may better reflect the extent of the limited diffusion of water molecules in tumor parenchyma. Previous research has also postulated that ADC images are more effective when attempting to identify tumor properties relative to the use of other morphological images such as T1WI and T2WI (35, 36). Recently, some researchers have applied radiomic technology to deeply dig into ADC images and found that they could not only evaluate tumor grading but also predict whether the tumor had metastasised (37, 38). This indicates that, acting as important MRI functional images, ADC images may show greater value in radiomic analysis than other sequences.

### The Combination of Radiomic and Clinical Indicators Further Improves the Accuracy of Prediction

Although Model^R^ showed a good level of discriminative ability, we still moved forward with adding the two commonly used clinical indicators BMI and CA125 to explore whether the diagnostic ability of this model could be further improved. It is a continuing trend in the development of radiomic technology to combine histochemical characteristics with clinical data to predict the degree of malignancy and the prognosis of tumors. It is well known that a high BMI is an independent risk factor for EC and it is suggested that the higher incidence of tumors in this context may be due to heightened oestrogen levels brought on by obesity (39). Previous studies have also surmised that the CA125 level in EC patients is higher than that in normal subjects, which may be because EC patients usually experience endometrial barrier breakdown, shedding, deformation and necrosis of trophoblastic cells and secretion of trophoblastic cells, which will increase the CA 125 level in peripheral blood (40). In this study, it was further discerned that a statistical difference between EC in the low- and high-risk patient populations exists. Therefore, we included these two indicators in Model^M^.

This study ultimately determined that the level of efficiency of Model^M^ for the differential diagnosis was higher than that of both Model^R^ and Model^ADC^. Of note, no statistically significant difference between Model^M^ and Model^R^ in the test cohort was observed, but this may be due to the small number of test cohort samples. A previous multi-sequence MRI radiomic analysis showed that MRI texture features are of high diagnostic value in predicting high-grade EC and LVSI, with accuracies of 80% and 70%, respectively (41). However, this previous study only considered radiomic indicators and failed to comprehensively assess clinical indicators; moreover, the sample size was small. The current study increased the sample size and combined clinical indicators to achieve an accuracy of 85% in distinguishing between the two risk tumors. Another prediction study comprehensively evaluated the predictive effect of a clinical, radiomic and mixed models on lymph node metastasis; finally, pathology analysis confirmed that mixed model had the strongest predictive effect on lymph node metastasis (18). Previous researchers have also used MRI texture analysis to predict the pathological risk grade and survival time of patients with EC. However, this approach only applies six first-order parameters and adopts two-dimensional image segmentation. Although the operation is simple and less time consuming, only a single-layer image of the diseased tissue can be obtained and it is difficult in this manner to fully reflect the tumor information (18). In the present study, large numbers of shape-based and second-order features were included, with more diverse properties. Meanwhile, 3D multi-layer segmentation was adopted such that variations in different risk levels of tumors were more likely to be found. At the same time, BMI and CA125 are added into the model and the training set AUC of the comprehensive nomogram was calculated to be about 0.925, indicating that the mixed model showed good predictive ability. Meanwhile, in the test cohort, the AUC of the comprehensive model reached 0.915, which confirmed that this model had good discriminative ability.

In addition, the sensitivity, specificity and accuracy of the radiomics based nomogram model (Model^M^) for EC risk classification were 88.8%, 81.5% and 84.9%, respectively. A previous study has found that using D&C, sensitivities for combined pre-operative testing for G3 endometrioid is only 56% and the specificity is 33%, both of which are lower than that in our study (42). Therefore, only histological procedures (curettage or biopsy) may not enough be the only method in the diagnosis of endometrial diseases. In terms of pathological grading of endometrial cancer, there may be inconsistency between the results of curettage and postoperative pathological results (43). Although almost all institutions perform D&C for examination of endometrial cancer, there is a weakness about using D&C for the diagnosis because this blind procedure might miss endometrial cancer. Therefore, this procedure has a high rate of false negatives, which is low at 51% (44). It has been reported that less than half of the uterine cavity is curetted in 60% of cases (45), and over 40% of women with complex atypical hyperplasia as a preoperative diagnosis have a final confirmation of endometrial cancer during hysterectomy (44, 46). In addition, a previous study has shown that postoperative pathological grading is elevated in some patients, that is, some endometrial cancers diagnosed as low pathological grade by preoperative curettage are proved to be high pathological grade by postoperative pathology (47). Another study found that 50 percent of 176 patients with endometrial carcinoma had an elevated pathological grade after surgery (48). Therefore, although D&C is still irreplaceable as the mainstream method of preoperative evaluation of EC, the comprehensive model based on radiomics proposed in this study for preoperative prediction of tumor properties may help to improve the accuracy of EC grading and make a supplement for the formulation of surgical plan.

This study has the following limitations that should be considered. First, this study is a retrospective study with small sample size and the reliability of its conclusions needs to be verified by a prospective study in the future. Second, the model established in this study relied on data from a single centre and a single MRI scanning system, so further multi-centre and multi-modality research should be explored. Finally, although the total number of samples in this study was large, the sample size in the test cohort was small and the difference between Model^M^ and Model^R^ could not be confirmed. Therefore, it is necessary to further expand the study sample size in the future.

In conclusion, by comparing the various models mentioned herein, we found that the mixed model based on the radiomic model was a good predictor of the histological risk grade of EC. In this study, several radiomics parameters based on ADC and venous phase images were strongly correlated with tumor risk grade. When tumor patients presented as overweight or obese together with an elevated CA125 level and a high radiomic score, they were more likely to be classified as high-risk cases. Using radiomic parameter-based model and nomogram analysis can help guide preoperative non-invasive grading of EC and avoid possible under- or overtreatment.

## Data Availability Statement

The raw data supporting the conclusions of this article will be made available by the authors, without undue reservation.

## Ethics Statement

The studies involving human participants were reviewed and approved by Qinhuangdao Municipal No. 1 Hospital. The ethics committee waived the requirement of written informed consent for participation.

## Author Contributions

TZ, LY, and LL designed and coordinated the study, TZ, JD, YD, SW, XW, and QS carried out experiment and data process, and drafted the manuscript. All authors contributed to the article and approved the submitted version.

## Funding

This research was supported by National Natural Science Foundation of China (81871029) Scientific Research Fund Project of Health Commission of Hebei Province (20200138).

## Conflict of Interest

Author QS was employed by company, Siemens Ltd.

The remaining authors declare that the research was conducted in the absence of any commercial or financial relationships that could be construed as a potential conflict of interest.
