# Supplementary material for: Combination Analysis of a Radiomics-Based Predictive Model With Clinical Indicators for the Preoperative Assessment of Histological Grade in Endometrial Carcinoma
Source: Front Oncol. 2021 Jun 21;11:582495. doi: 10.3389/fonc.2021.582495 (PMC8255911; doi:10.3389/fonc.2021.582495)
Supplement: Supplementary file 1 [file DataSheet_1.docx]

Supplementary Methods

1. Supplementary Method S1. Radiomic features

In this study, a total of 100 candidate radiomic features were generated from axial T2WI FS-FSE, axial ADC and arterial, venous and delayed phase of 3D-ISO-VIBE, including 500 texture features for each candidate.

All radiomic features could also be calculated from derived images, obtained by applying a variety of filters. The derived images usually showed greater ability in pattern classification. All features defined below are in compliance with feature definitions as described by the Imaging Biomarker Standardization Initiative (IBSI), which are available in a separate document by Zwanenburg et al. (2016) [[1]](https://pyradiomics.readthedocs.io/en/latest/features.html#id3).

**The shape-based features** count a number of features of descriptors of the three-dimensional size and shape of the ROI. These features are independent from the gray level intensity distribution in the ROI and are therefore only calculated on the non-derived image and mask. Unless otherwise specified, features are derived from the approximated shape defined by the triangle mesh. To build this mesh, vertices (points) are first defined as points halfway on an edge between a voxel included in the ROI and one outside the ROI. By connecting these vertices a mesh of connected triangles is obtained, with each triangle defined by 3 adjacent vertices, which shares each side with exactly one other triangle. The basic shape voxel volume statistics features include 14 features: VoxelVolume, Maximum3DDiameter, MeshVolume, MajorAxisLength, Sphericity LeastAxisLength, Elongation, SurfaceVolumeRatio, Maximum2DDiameterSlice, Flatness, SurfaceArea, MinorAxisLength, Maximum2DDiameterColumn, Maximum2DDiameterRow.

**First-order statistics features** describe the distribution of voxel intensities within the image region defined by the mask through commonly used and basic metrics. The basic first-order statistics features include Energy, Total Energy, Entropy, Minimum, 10th percentile, 90th percentile, Maximum, Mean, Median, Range, Mean Absolute Deviation (MAD), Robust Mean absolute Deviation (rMAD), Root Mean Squared (RMS), Skewness, Kurtosis, Variance, and Uniformity, totally 18 features.

**Second-order statistics features.** In image analysis, texture is one of the deﬁning sets of features. Texture features were originally designed to assess surface texture in 2D images. Texture analysis is however not restricted to 2D slices and can be extended to 3D objects. In this study Gray Level Co-occurrence Matrix (GLCM), Gray Level Dependence Matrix (GLDM), Gray Level Run Length Matrix (GLRLM) Gray Level Size Zone (GLSZM) are included.

**The grey level co-occurrence matrix (GLCM)** is a matrix that expresses how combinations of discretised intensities (grey levels) of neighbouring pixels, or voxels in a 3D volume, are distributed along one of the image directions. Generally, the neighbourhood for GLCM is a 26-connected neighbourhood in 3D and a 8-connected neighbourhood in 2D. Thus, in 3D there are 13 unique direction vectors within the neighbourhood for Chebyshev distance. The basic GLCM statistics features include 22 features: Joint Average, Joint Entropy, Cluster Shade, Maximum Probability, Idmn, Joint Energy, Contrast, Difference Entropy, Inverse Variance, Difference Variance, Idn, Idm, Correlation, Autocorrelation, Sum Entropy, Sum Squares, Cluster Prominence, Imc2, Imc1, Difference Average, Id, Cluster Tendency.

**Gray Level Dependence Matrix (GLDM)** quantifies gray level dependencies in an image. A gray level dependency is defined as a the number of connected voxels within distance δ that are dependent on the center voxel. A neighbouring voxel with gray level jj is considered dependent on center voxel with gray level ii if |i−j|≤α|i−j|≤α. In a gray level dependence matrix P(i,j)P(i,j) the (i,j)(i,j)th element describes the number of times a voxel with gray level ii with jj dependent voxels in its neighbourhood appears in image. The basic GLCM statistics features include 14 features: Gray Level Variance, High Gray Level Emphasis, Dependence Entropy, Dependence Non-Uniformity, Gray Level Non-Uniformity, Small Dependence Emphasis, Small Dependence High Gray Level Emphasis, Dependence Non-Uniformity Normalized, Large Dependence Emphasis, Large Dependence Low Gray Level Emphasis, Dependence Variance, Large Dependence High Gray Level Emphasis, Small Dependence Low Gray Level Emphasis, Low Gray Level Emphasis.

**The grey level run length matrix (GLRLM)** was introduced to define various texture features. Like the grey level co-occurrence matrix, GLRLM also assesses the distribution of discretised grey levels in an image or in a stack of images. However, whereas GLCM assesses co-occurrence of grey levels within neighbouring pixels or voxels, GLRLM assesses run lengths. The basic GLRLM statistics features include 16 features: Gray Level Non-Uniformity, Gray Level Non-Uniformity Normalized, Gray Level Variance, High Gray Level Run Emphasis, Long Run Emphasis, Long Run High Gray Level Emphasis, Long Run Low Gray Level Emphasis, Low Gray Level Run Emphasis, Run Entropy, Run Length Non-Uniformity, Run Length Non-Uniformity Normalized, Run Percentage, Run Variance, Short Run Emphasis, Short Run High Gray Level Emphasis, Short Run Low Gray Level Emphasis.

**The grey level size zone matrix (GLSZM)** counts the number of groups (or zones) of linked voxels. Voxels are linked if the neighbouring voxel has an identical discretised grey level. Whether a voxel classifies as a neighbour depends on its connectedness. In a 3D approach to texture analysis we consider 26-connectedness, which indicates that a center voxel is linked to all of the 26 neighbouring voxels with the same grey level. In the 2 dimensional approach, 8-connectedness is used. A potential issue for the 2D approach is that voxels which may otherwise be considered to belong to the same zone by linking across slices, are now two or more separate zones within the slice plane. Whether this issue negatively affects predictive performance of GLSZM-based features or their reproducibility has not been determined. The basic GLZSM statistics features include 16 features: Gray Level Non-Uniformity, Gray Level Non-Uniformity Normalized, Gray Level Variance, High Gray Level Zone Emphasis, Large Area Emphasis, Large Area High Gray Level Emphasis, Large Area Low Gray Level Emphasis, Low Gray Level Zone Emphasis, Size Zone Non-Uniformity, Size Zone Non-Uniformity Normalized, Small Area Emphasis, Small Area High Gray Level Emphasis, Small Area Low Gray Level Emphasis, Zone Entropy, Zone Percentage, Zone Variance.

After the procedure of feature selection, three features was used to fit the model and calculate the Radscore. They are LargeDependenceLowGrayLevelEmphasis@venous, Maximum2DDiameterColumn@ADC, LowGrayLevelZoneEmphasis@ADC. Large Dependence Low Gray Level Emphasis (LDLGLE) measures the joint distribution of large dependence with lower gray-level values. The specific calculation formula is as follows (1-1). Maximum 2D diameter (Column) is defined as the largest pairwise Euclidean distance between tumor surface mesh vertices in the row-slice (usually the coronal or sagittal) plane. Low Gray Level Zone Emphasis (LGLZE) measures the distribution of lower gray-level size zones, with a higher value indicating a greater proportion of lower gray-level values and size zones in the image. The specific calculation formula is as follows (1-2).

$LDLGLE=\frac{\sum_{i=1}^{N_{g}} \sum_{j=1}^{N_{d}} \frac{\mathbf{P}\left( \boldsymbol{i,j} \right)\boldsymbol{j}^{\boldsymbol{2}}}{i^{2}}}{N_{z}}$ （1-1）

***where***

N_g_ is the number of discreet intensity values in the image

N_d_ is the number of discreet discreet dependency sizes in the image

N_z_ is the number of dependency zones in the image, which is equal to $\sum_{i=1}^{N_{g}} \sum_{j=1}^{N_{d}} \mathbf{P}\left( i,j \right)$

**P**(i,j) is the dependency matrix

$LDLGLE=\frac{\sum_{i=1}^{N_{g}} \sum_{j=1}^{N_{s}} \frac{\mathbf{P}\left( \boldsymbol{i,j} \right)}{i^{2}}}{N_{z}}$ （1-2）

***where***

N_g_ is the number of discreet intensity values in the image

N_s_ is the number of discreet zone sizes in the image

N_p_ is the number of voxels in the image

N_z_ is the number of zones in the ROI, which is equal to $\sum_{i=1}^{N_{g}} \sum_{j=1}^{N_{r}} \mathbf{P}\left( i,j \right)$ and 1≤N_z_≤N_p_

**P(i,j)** is the size zone matrix

1. Supplementary Method S2. Normalization and prepropocess of the features

The feature matrix was normalized with the "Normalization" module of FAe software. In order to explore the correlation between different features and label, it is necessary to scale the values of different features directly to the same order of magnitude, that is, to process the values of all cases corresponding to each feature. In this study, the method of "Normalize to unit" was used for normalization. The specific calculation formula is as follows (2-1).

$\vec{x_{n}}=\frac{\vec{x_{n}}-\bar{x_{n}}}{\sqrt{x_{1n}^{2}+x_{2n}^{2}+\ldots+x_{mn}^{2}}}$ (2-1)

***where***

$\vec{x_{n}}$ is the 𝓃’th feature， m is the number of samples.

In this study, the number of radiomics features is much larger than the number of samples. In order to conduct efficient feature selection and model building, the “preoperation” module of FAE can preprocess the data and reduce the number of features. In this study, Pearson Correlation Coefficients (PCC) were used to conduct dimensionality reduction processing on the data. This method traverses all the eigenvalues and calculates Pearson correlation coefficient in pairs. When the coefficient is larger than the threshold value (currently the default is 0.86), one of them is removed randomly. This method can make the features after dimensionality reduction do not have high similarity. The specific calculation formula is as follows (2-2).

$\cos\theta_{12}=\frac{\vec{x_{1}}\cdot\vec{x_{2}}}{|\vec{x_{1}}||\vec{x_{2}}|}$ (2-2)

[[1]](https://pyradiomics.readthedocs.io/en/latest/features.html#id3)Zwanenburg, A., Leger, S., Vallières, M., and Löck, S. (2016). Image biomarker standardisation initiative - feature definitions. In eprint arXiv:1612.07003 [cs.CV]

1. Supplementary Table

**TABLE 1**: Performances of the models in the training cohort

| Model | AUC (95% CI) | Sensitivity (%) | Specificity (%) | Accuracy (%) |
| --- | --- | --- | --- | --- |
| Model^ADC^ | 0.715 (0.651-0.779) | 61 | 74.2 | 68 |
| Model^R^ | 87(82.8-91.3) | 72 | 85.6 | 79.2 |
| Model^M^ | 0.925(0.898-0.951) | 88.8 | 81.5 | 84.9 |

**TABLE 2**: Performances of the models in the test cohort

| Model | AUC (95% CI) | Sensitivity (%) | Specificity (%) | Accuracy (%) |
| --- | --- | --- | --- | --- |
| Model^ADC^ | 0.621 (0.515-0.726) | 43.1 | 79 | 62 |
| Model^R^ | 89.2(83.3-95.1) | 76.5 | 89.5 | 83.3 |
| Model^M^ | 0.915(0.863-0.968) | 92.2 | 79 | 85.2 |

1. Supplementary Figure


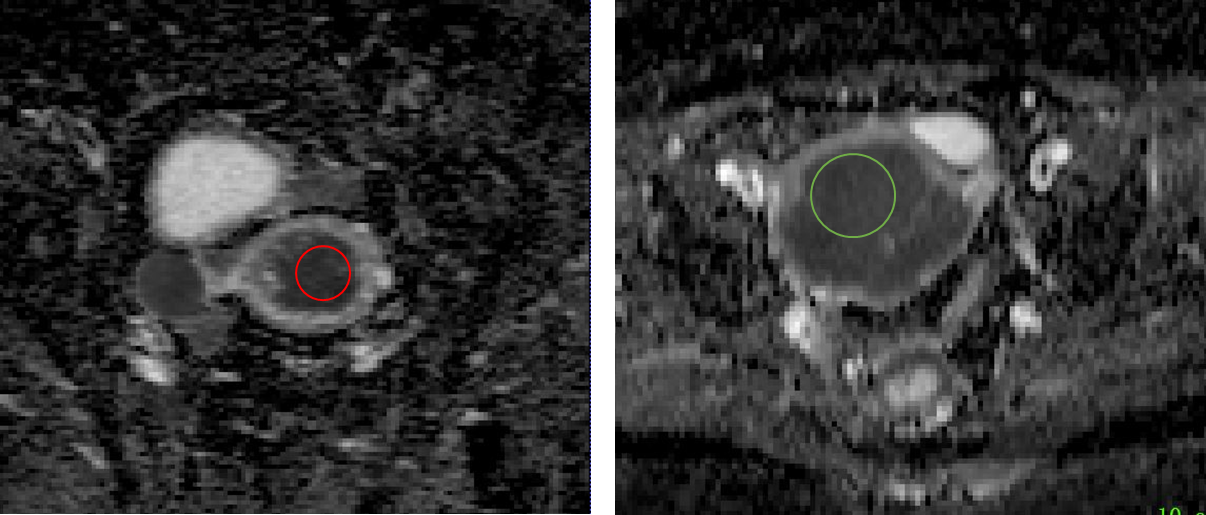


Supplementary Figure 2: The tumor ROI was outlined on the ADC maps. (A) ADC map of a 56-year-old female with EC. The pathological grade was confirmed by surgery as G1. The red circle is a hand-sketched ROI with an ADC value of 1.3×10 ^-3^ mm^2^/s. The area of the ROI is 3.1 cm^2^.(B) ADC map of a 67-year-old female with EC. The pathological grade was confirmed by surgery as G3. The green circle is a hand-sketched ROI with an ADC value of 0.9×10 ^-3^ mm^2^/s. The area of the ROI is 11.7 cm^2^.
